# Massachusetts Dental Society, Annual Meeting

**Published:** 1902-07

**Authors:** 


### MASSACHUSETTS DENTAL SOCIETY, ANNUAL MEETING.

The thirty-eighth annual meeting of this Society was held at Hotel Brunswick, Boston, on Wednesday and Thursday, June 4 and 5, 1902, and the writer, for the first time, had the pleasure of mingling with Massachusetts men in convention. To assert that it is always pleasant to associate with those of our own household is a truism, but in this case it was doubly gratifying to find that the men of Massachusetts were not only of our own household, but they had absorbed the spirit which properly enters into the development of true professional life.

The meeting was attended by a large number, the membership being nearly doubled upon this occasion.

The papers were of interest. The first was "Crown- and Bridge-Work to the Country Dentist," by Dr. Walter F. Bisbee, of Camden, Me. Dr. Horace E. Eaton, of Toronto, Canada, undertook to solve the difficult problem of how to handle children in the dental office. Those who expected a detail of old familiar methods were agreeably surprised to find that the Eaton system had not only a flavor of originality, but in his hands it seemingly solved a great professional difficulty. It is questionable, however, whether many will be able to adopt his very sensible ideas.

Dr. E. C. Kirk, of Philadelphia, gave a description of his investigations with the oral fluids, using the projecting micropolariscope to illustrate.

Dr. Joseph Head, of Philadelphia, and Dr. Loomis P. Haskell, of Chicago, the first on "Porcelain Inlays" and the latter on "Prosthetic Dentistry," with models, were followed by Dr. J. N. Crouse, on "The Present Condition of the Dental Protective Association." These formed the principal features, in papers, of the first and second days. The clinics covered a variety of subjects, and possessed a practical value to those who can see the operations, but this difficulty of not being able to see will always be an objection to these public efforts.

The meetings were all held at the Hotel Brunswick, and everything was done by the proprietors of that house to make things pleasant for every one.

The centre of attraction was the banquet held Wednesday evening. A novel feature, and believed to be peculiar to New England, was the making this "Ladies' Night." Their presence contributed much to the enjoyment of the occasion. The usual accompaniment of gastronomic efforts composed exclusively of men, wine, and cigars, were notable by their absence. While the inner man was well supplied, the chief attractions were musical and intellectual.

Before the President, Dr. Frederick E. Faxon, called to order, the tables in the centre of the room were all dexterously removed by the waiters, and a true social gathering of two hundred earnest men and women were grouped together to consider the important question of "The Law in Relation to the Practice of Dentistry." It was an unusual thing to discuss a serious question, such as this, at a banquet, but, notwithstanding it was unusual and not a very entertaining subject, it held the audience to a late hour.

The speakers, it is presumed, had no idea of settling this much controverted question, but they undoubtedly endeavored to help thought in the direction in which they were interested.

The law side of the question was explained by Hon. James A. McGeough, and the dental side, for and against, was energetically discussed by Drs. Kirk, Guilford, Head, Parmelee, Hurlburt, Fillebrown, and Truman. Possibly the laws will remain on the statute books notwithstanding this avalanche of words, but, aside from this, it proved a delightful change from the ordinary tedious after-dinner talks that have become too much the dread of speakers and auditors. Massachusetts has set an example that others can follow with marked advantage.

The music of the occasion was unique in some of its features. That this was an enjoyable part of the programme was to have been expected in Boston, the centre of musical training in this country, but a peculiar addition was that each speaker was introduced by music and a song. The effect was as excellent as it was novel. It was late when the banquet broke up, with the feeling by the writer that the hours had been most pleasantly and not unprofitably spent.

Massachusetts men made a great effort to have this meeting notable among the thirty-eight annual gatherings, and that they succeeded those who were honored as guests can most truly affirm, and for the writer it was an occasion to be remembered for many things, but most of all for that advanced professional spirit manifested throughout, and which must make a decided impression upon the succeeding decades of the twentieth century.

